# Can microfluidics be used to create a more realistic in vitro model of the vaginal ectocervix to better understand bacterial vaginosis?

**DOI:** 10.1136/sextrans-2021-055225

**Published:** 2021-09-08

**Authors:** Ashley Houlden, Ruth Mackay

**Affiliations:** 1 Biomedical Sciences, College of Health, Medicine and Life Sciences, Brunel University London, Uxbridge, UK; 2 Mechanical and Aerospace Engineering, College of Engineering, Design and Physical Sciences, Brunel University London, Uxbridge, UK

**Keywords:** vaginosis, bacterial, microbiology, women

Bacterial vaginosis (BV) is an under-reported, underdiscussed and under-researched condition despite its high prevalence of 12%–50% worldwide.^1^ BV is characterised by an overgrowth of anaerobic bacteria, originally attributed to *Gardnerella vaginalis* (GV), disrupting the typically dominant lactobacilli vaginal ecosystem, resulting in discomfort and vaginal discharge with fishy odour. Women with BV have higher risk of transmission of STI and poor perinatal outcome.^1^ The aetiology of BV remains unknown, with a large number of bacteria associated to the condition and GV presence showing variable meaning. BV is a multifactorial syndrome involving the interaction between the host vaginal microbial ecosystem and environmental factors. Despite treatment with metronidazole (used for the past 25 years), up to 50% of cases reoccur within 12 months.

In vitro models have been developed, including two-dimensional, organoid and animal models; however, researchers are calling for a more representative model to investigate BV.^1^ Over the past decade a new technology to replicate three-dimensional tissues has been established by microfluidic researchers, organ-on-a-chip (OOC). These in vitro models use microfluidic chips embedded with a scaffold and cells from specific organs. Using organ-specific forces and fluidic shear flow these micro-tissues can represent the physiological environment of the organ under investigation. Numerous OOC models have been developed (lung, gut, heart and brain). OOC gut experiments have included bacterial seeding to mimic the microbiome on top of Caco-2 cells cultured to form phenotypical intestinal villi.^2^ While some groups are looking at female reproductive organs, no one has developed an OOC model of the vaginal ectocervix.

BV is a common condition that requires greater understanding and new treatments. OOC gives researchers in the field a way forward to create a model that represents the vagina in a way not seen before.
